# A Review of the Pharmacological Action of Astragalus Polysaccharide

**DOI:** 10.3389/fphar.2020.00349

**Published:** 2020-03-24

**Authors:** Yijun Zheng, Weiyu Ren, Lina Zhang, Yuemei Zhang, Dongling Liu, Yongqi Liu

**Affiliations:** ^1^Provincial-level Key Laboratory for Molecular Medicine of Major Diseases and The Prevention and Treatment with Traditional Chinese Medicine Research in Gansu Colleges and University, Gansu University of Chinese Medicine, Lanzhou, China; ^2^Pharmacy College, Gansu University of Chinese Medicine, Lanzhou, China; ^3^School of Education, University of Leeds, Leeds, United Kingdom; ^4^Ophthalmology Department, First Hospital of Lanzhou University, Lanzhou, China

**Keywords:** Astragalus polysaccharide, preparation, chemical composition, pharmacological action, immune regulation, anti-aging, anti-tumor, regulation of blood glucose

## Abstract

*Astragalus membranaceus* (*A. membranaceus*) is a type of traditional Chinese medicine with a long history of clinical application. It is used in the improvement and treatment of various diseases as medicine and food to invigorate the spleen and replenish qi. The main components of *A. membranaceus* are Astragalus polysaccharide (APS), flavonoids compounds, saponins compounds, alkaloids, etc. APS is the most important natural active component in *A. membranaceus*, and possesses multiple pharmacological properties. At present, APS possess the huge potential to develop a drug improving or treating different diseases. In this review, we reveal the potential approaches of pre-treating and preparation on APS as much as possible and the study on content of APS and its chemical composition including different monosaccharides. More importantly, this paper summarize pharmacological actions on immune regulation, such as enhancing the immune organ index, promoting the proliferation of immune cells, stimulating the release of cytokines, and affecting the secretion of immunoglobulin and conduction of immune signals; anti-aging; anti-tumor by enhancing immunity, inducing apoptosis of tumor cells and inhibiting the proliferation and transfer of tumor cells; antiviral effects; regulation of blood glucose such as type I diabetes mellitus, type II diabetes mellitus and diabetic complications; lipid-lowering; anti-fibrosis; antimicrobial activities and anti-radiation. It provided theoretical basis for the further research such as its structure and mechanism of action, and clinical application of APS.

## Introduction

*A. membranaceus* is one of the most popular herbal medicines worldwide. In China, it is known as “Huangqi.” In traditional Chinese medicine, it is used as medicine and food to invigorate the spleen and replenish qi. Astragalus polysaccharide (APS) is a type of water-soluble heteropolysaccharide with bioactive effects, which is extracted from the stems or dried roots of *A. membranaceus*. The components are complex and diverse, and polymeric carbohydrates are mainly linked by α-type glycosidic bonds between the monosaccharides (Li C. et al., 2019). APS is the most important natural active component in *A. membranaceus* and exerts multiple pharmacological effects (Li S.S. et al., 2019). Owing to its low toxicity and side effects, non-residue, and non-tolerance, APS has been widely utilized (Zhang and Zhu, 2010). In this review, the authors examined the extraction and structural composition of APS, as well as the effects on immune function in immune organs, cells, and molecules. Furthermore, the latest research progress on the pharmacological effects of APS was illustrated to provide the theoretical basis for the clinical application of APS.

## Preparation of APS

APS, one of the natural active components in *A. membranaceus*, is an active substance which is beneficial to human health. Thus far, the extraction methods of APS mainly involve water extraction, microwave extraction, enzyme extraction, alkali water extraction, etc. The purity of APS extracted at the same dose varies because of the different preparation methods. Studies showed that the best extractive method of APS by water is to perform the extraction twice through heating water at 100°C for 60 min per extraction. The liquid ratio of the material is 1:10 g/ml, and the rate of extraction is 3.570%. The use of tannic acid to split protein provides the best effect. Moreover, the purification of APS, which is isolated and purified by D101 macroporous absorptive resin, is 65.07% (Liu et al., 2018). The water extraction method will extract other substances, such as saponins and flavonoids, from *A. membranaceus*. These substances are difficult to separate at the later stage, resulting in the low purity of APS. Moreover, the energy consumption of this method is high, whereas the economic benefit is relatively low. However, the extraction ratio of APS was significantly improved with the alkali extraction method versus water extraction (Li et al., 2000). Alkali destroy the fiber of *A. membranaceus*, facilitating the flow of polysaccharides. Compared with the method of APS extraction using heated water, microwave-assisted extraction reduced the amount of extractant, shortened the extraction time, and increased the production of APS (Gong and Yang, 2004). Microwaves can inactivate the enzymes in the cell membrane and cell wall. This facilitates the flow of APS, increasing the yield of polysaccharides. Similarly, it was found that the extraction rate can reach 92.1% after 1 h of ultrasonic extraction through the method of ultrasonic-assisted extraction of APS (Song and Zhao, 2010). Cellulose is the main component of the cell wall. Cellulase can destroy the cell wall of *A. membranaceus* and improve the extraction rate of polysaccharide. Following the extraction of APS using the cellulase method, the content of polysaccharide was 9.78% and the total sugar content was 50.2% (Chen and Ma, 2005). The extraction of APS using the alcohol alkali method resulted in 3.53-fold and 2.63-fold higher yield compared with the water extraction and alkali extraction methods, respectively (Tian et al., 2006). When APS was extracted using a calcium oxide solution, the yield of APS was different under different conditions. The results showed that the yield and purity of the extracted APS was highest when pH was 9.0 (Liu et al., 2010). Furthermore, other studies showed that the new methods of pressurized liquid extraction, microwave-assisted acidic hydrolysis, and comprehensive chromatography are more effective, more suitable for carbohydrate analysis, and can more optimally control the quality of APS extraction (Lv et al., 2015). The extraction of APS through homogenization-assisted negative pressure cavitation extraction could increase the yield of polysaccharides to approximately 15% (Jiao et al., 2014). In addition, the methods of extracting APS also include ultrahigh pressure technology extraction, rapid extraction with a high-voltage pulsed electric field, etc. (Liu et al., 2009). The extraction methods for plant polysaccharides also include using a response surface methodology on Box-Behnken design (Zhao C. et al., 2017), application of ammonia fiber expansion pretreatment (Qiao et al., 2016; Zhao et al., 2016; Zhao L.C. et al., 2017), etc. Astragalus contains water, and the determination of water content should be performed before extraction of polysaccharides. Near-infrared spectroscopy technology can be used for testing on a wide range of water contents of plants (Zhang et al., 2019). Different extraction methods influence the composition of APS, while different structures of APS also influence its function. Through water boiling and alcohol precipitation, two components of APS, termed APS-I (sugar content: 94.59%) and APS-II (sugar content: 97.57%), are obtained by graded precipitation with 30% and 70% ethanol, respectively. By comparing the inhibitory effects on mice ascites tumors, it was found that the inhibitory rate of APS-I (50 mg/kg) was 55.47%, and the effect was higher than that of APS-II (50 mg/kg) by 47.72% (Zhu et al., 2011). Recent studies have found that the contents and functions of extracted APS at different temperatures are different. Although the main component of APS4 and APS90 extracted at 4°C and 90°C is glucose and the main chain is composed of (1→2) α-D-Glcp, APS4 shows a higher content of (1→2) α-D-Glcp and exerts the greatest inhibitory effect on MGC-803, A549, and HepG2 cells. This indicates that the higher branching degree may be responsible for the strong *in vitro* antitumor activity of APS4 (Yu et al., 2019). At present, the representative preparation of APS for injection (Chinese medicine standard Z20040086) is mainly used to improve immunity and assist in the treatment of diseases, such as cancer and diabetes (Zheng et al., 2013).

## Study on Chemical Composition of APS

APS is the macromolecular substance and its structure material contains numerous carbohydrates. The content of APS in *A. membranaceus* is different depending on the medicinal part, basic sources, place of production, planting method, and growth year. Some studies showed that the polysaccharide content in stems and leaves of *Astragalus mongholicus Bge* (*A. mongholicus*) is far lower than that in roots (Bi et al., 2017). By comparing the quality of *A. membranaceus* and *A. mongholicus* using metabolomics, it was found that the content of mannose, xylose, and other soluble sugars in the former was significantly higher than that noted in the latter (Duan et al., 2012). Among the four places of production in China (i.e., Heilongjiang, Inner Mongolia, Shanxi, and Gansu), the Gansu area yields the highest content of APS (Zhu et al., 2016). The content of polysaccharides and glycoconjugates in wild *A. membranaceus* is generally higher than that measured in fast-growing *A. membranaceus* (Li et al., 2015). Some studies have measured the content of APS in 1–3 years *Astragali radix*. They found that the content of polysaccharide in 1-year *Astragali radix* is the highest, and the content of polysaccharide gradually decreases with time (Zhang et al., 2006). This is attributed to the complex chemical structures and content differences in polysaccharides. Hence, it is relatively difficult to separate or represent each component. The polysaccharides isolated from *A. membranaceus* are mostly extracted in the form of white powder; some studies showed that the relative molecular weight of APS was 10–50 kDa (Jiang et al., 2013).

Due to the complicated chemical structure of polysaccharide, the cognition on accurate components of APS is very limited. The monosaccharide composition and proportion of polysaccharides with different molecular weights are different. Hence, the sugar chain connection sequence and glycosidic bond types, as well as the corresponding biological activities will also differ. There are eight main glycosidic bond types of APS measured through gas chromatography–mass spectrometry. Of those, 1,4- glucose linkage is the main, and nuclear magnetic resonance confirmed that anomeric hydrogen is characterized by α configuration (Wang et al., 2017). The main components of APS include heteropolysaccharide, dextran, neutral polysaccharide, and acidic polysaccharide. Heteropolysaccharide is an acidic water-soluble polysaccharide, while dextran is divided into water-soluble and water-insoluble forms (Gao and Gao, 2017), namely α(1→4)(1→6) dextran and α(1→4) dextran, respectively (Chen and Huang, 2008). A type of acid heteropolysaccharide is isolated from the root of *A. mongholicus* and its relative molecular weight is 76 kDa. It is composed of l-arabinose-d-galactose-d-galacturonic acid-d-glucuronic acid (18:18:1:1), a small quantity of O-acetyl groups, and peptide residue (Ai et al., 2008). Studies also showed that there were six types of monosaccharides isolated from APS, namely amylaceum, seminose, arabinose, xylose, glucuronic acid, and rhamnose; the composition ratio of these monosaccharides (nmol) was 12.83: 0.27: 0.71: 1.63: 1.04: 0.56, respectively (Liao et al., 2018). Of the 14 types of polysaccharides isolated from Astragalus, 13 have β-d(1→6)-galactooligosaccharide branching β-D-(1→3)-galactose (Kiyohara et al., 2010). In total, there are 24 types of polysaccharides extracted from the root of *A. membranaceus*, and most of them are heteropolysaccharides. The molecular weight of heteropolysaccharides ranges 8.7–4,800 kDa; they are composed of different monosaccharides, including l-rhamnose, l-rabinose, d-xylose, l-xylose, d-ribose, l-ribose, d-galactose, d-glucose, and d-mannose (Jin M. et al., 2013).

## Pharmacological Action of APS

**Figure 1:**
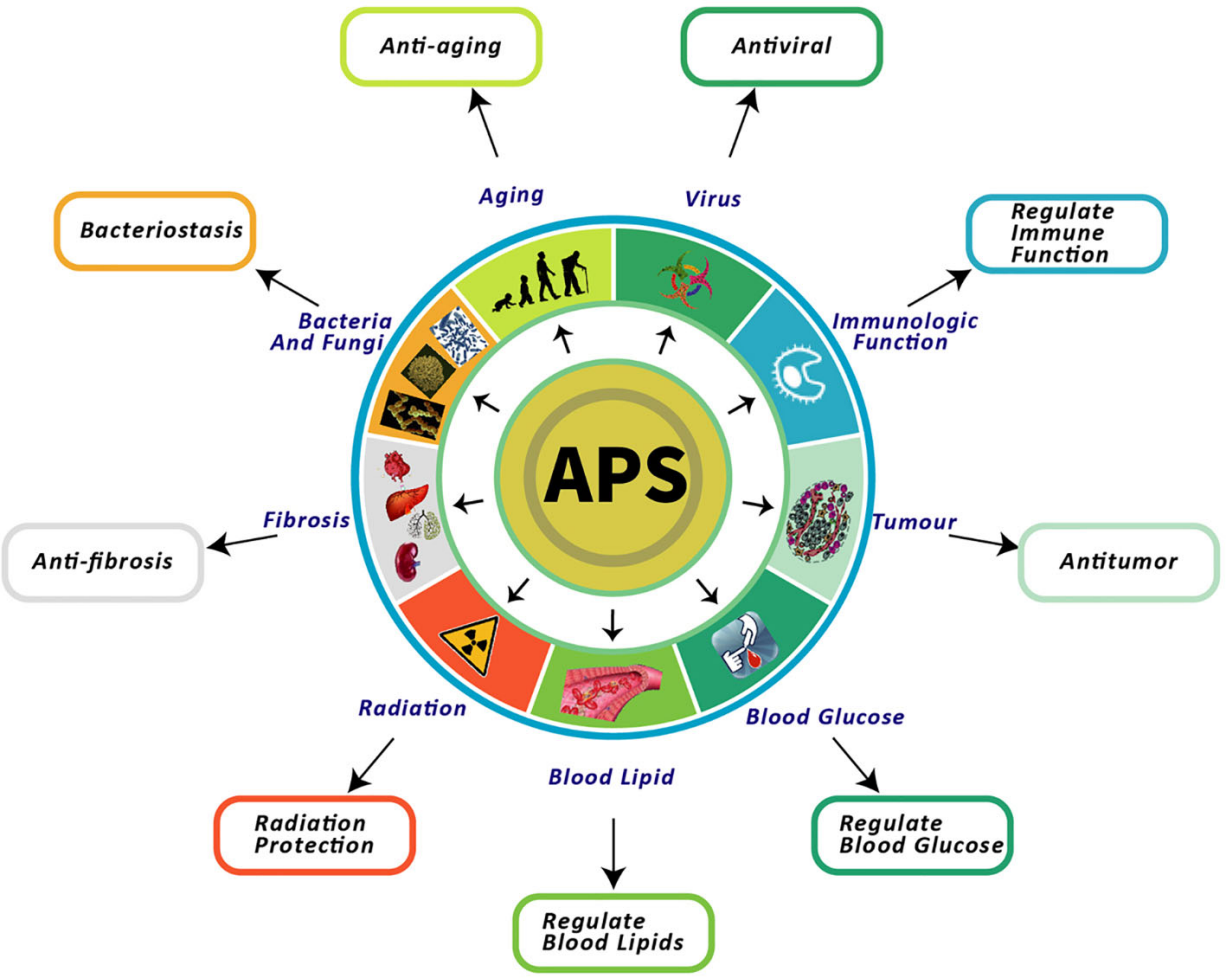
Nine pharmacological effects of APS. This figure shows the most highlighted nine effects in studies on APS.

APS exerts multiple pharmacological effects. In particular, nine of these effects have been thoroughly investigated, including the regulation of immune function, and anti-aging, antitumor, reducing blood sugar, lowering blood lipid, anti-fibrosis, antibacterial, radiation protection, and antiviral effects (**Figure 1**).

### Regulation Effect of APS on Immunity

APS regulates the immune function by enhancing the immune organ index, promoting the proliferation of immune cells, stimulating the release of cytokines, and affecting the secretion of immunoglobulin (Ig) and conduction of immune signals.

#### Influences on Immune Organs

Many studies showed that APS can act on various immune organs to increase organ weight, improve organ index, and promote the development of partial visceral organs. In a study investigating the effect of APS on H22 tumor-bearing mice, it was found that fluorouracil significantly inhibited tumor growth; the thymus was obviously degenerated, and the spleen was obviously swollen. After treatment with APS, the thymus and spleen showed obvious improvement compared with those observed in the model group (Yu et al., 2018). Recent studies showed that after treatment with APS in Lewis transplantable lung cancer mice, the spleen and thymic immune organ indices in APS group were higher than those determined in normal saline group. This effect may improve the function of immune organs by reducing the expression of vascular endothelial growth factor and epidermal growth factor receptor, thereby inhibiting the tumor. (Zhao et al., 2019). Similarly, the APS+polysaccharopeptide herbal formulation significantly improved the immune function of mice with Lewis lung cancer and increased the spleen and thymus indices (Zhou et al., 2018). Besides, APS antagonized thymus atrophy in rats with incremental load training (Hu and Gao, 2011). When combined with probiotics, APS markedly improved the spleen, bursa of fabricius, and thymus index in Gushi chicken, especially in chicks. APS was able to promote the maturation of immune organs (Zhao et al., 2012), and enhanced the immune function by increasing the weight of immune organs.

#### Influences on Immune Cells

The influences of APS on immune cells mainly include an increase the proliferation and differentiation of B lymphocytes and T lymphocytes, regulation of the balance in the T lymphocyte subgroup, and regulation of natural killer cells and macrophages. Dendritic cells (DCs) are key in activating the immune response. Studies have shown that APS facilitates the growth and maturation of DCs and their antigen-presenting capacity, as well as decreases the endocytosis activity of DCs. Moreover, APS can markedly promote the development and maturation of bone marrow-derived DCs (Shao et al., 2006). APS activates T cells by inducing the differentiation of DCs (Liu et al., 2011). A recent study found that APS is the first effective regulator of tumor M1/M2 macrophage polarization and an effective activator of DC maturation (Bamodu et al., 2019). APS activates the proliferation of B cells and macrophages and increases the production of cytokines. Furthermore, it can activate B lymphocytes through antigen receptors on B cell membranes rather than through the toll-like receptor 4 (TLR4) (Shao et al., 2004). An *in vivo* study of mice revealed that APS is able to increase complement (C3) deposition and the number of macrophages (Wang et al., 1989). In addition, some studies have shown that APS promotes humoral immune response by regulating the functional activity of natural killer and natural killer T cells (Xie et al., 2013).

#### Influences on Immune Molecules

##### Influences on Cytokines

APS exerts different effects on cytokines under different conditions. Under normal physiological conditions, it can promote cytokine production and enhance immunity. However, following an increase in cytokines as a result of an inflammatory response, APS can reduce inflammatory response factors and protect cells or the body (**Table 1**). Studies have shown that APS affects the secretion and production of cytokines; it is able to promote splenocytes to produce interleukin 2 (IL2), induce interferon (IFN), and promote the secretion of IL3, IL4, and IL6 (Deng et al., 2012). Recent studies have found that adenosine monophosphate (AMP), as a potential immunomodulator, improves the serum levels of IL11, tumor necrosis factor-α (TNF-α), and IFNγ, and enhances spermatogenesis and sperm quality in mice (Qiu and Cheng, 2019). APS promotes immune regulation by inducing the production of IL in human body. Following the application of APS, the production of IL10, IL12, and IL2 was found to be dose-dependent compared with the negative control (Yin et al., 2012). Besides, APS can upregulate the expression of TNF-α, lysozyme C, and IL1β in the spleen, gill, and kidney of the carp, which was also found to be dose-dependent (Yuan et al., 2008). APS can also improve the expression of IL2 and IL10 in the jejunum of cyclophosphamide broilers (Li S. et al., 2019). It has been shown that the levels of TNF-α, IL6, and IL1β were significantly increased in mice with colitis induced by saline in *in vivo* studies. However, the production of these inflammatory cytokines was markedly decreased in the groups treated with APS and dexamethasone treatment group (Lv et al., 2017). Another study of experimental colitis in rats treated with APS found that A high dose of APS (200 mg/kg) or dexamethasone could markedly downregulate the expression of IL1β and TNF-α and upregulate the protein expression of nuclear factors of activated T cells 4 mRNA. Although a low dose of APS (100 mg/kg) markedly downregulated IL1β, it had no significant impact on the expression of TNF-α protein (Yang et al., 2014). *In vitro* studies have investigated the effect of APS on the inflammatory reaction in lipopolysaccharide-infected (LPS-infected) Caco-2 cells. The results showed that APS could significantly downregulate the expression of TNF-α, IL1β, and IL8 in Caco-2 cells infected with LPS (Wang et al., 2013). APS also restrained the expression of TNF-α and IL1β by inhibiting the activation of nuclear factor kappa-B (NF-κB) in THP-1 macrophages, which is induced by LPS (Liu et al., 2017). After stimulating RAW264.7 cells with LPS or APS for 24 h, the levels of TNF-α and IL6 were significantly increased. It was found through reverse transcription-polymerase chain reaction that the mRNA expression of IL6, TNF-α, and inducible nitric oxide synthase was strongly increased after treatment with APS (Li et al., 2018). A study of tumor-bearing mice found that APS could enhance the immune function by increasing the levels of cytokines, such as IL2, IL6, IL12, and TNF-α (Xiao et al., 2009). In a similar study, APS upregulated the expression of TNF-α, IL12, and IL2, whereas it decreased the levels of IL10 and downregulated the expression of multidrug resistance 1 mRNA and P-glycoprotein in H22 tumor-bearing mice (Tian et al., 2012; Yang et al., 2013). Another study revealed that APS can inhibit tumorous growth in tumor-bearing mice. The mechanism of this process may involve increasing the levels of TNF-α and IFNγ, and reducing those of IL10 and transforming growth factor-β (TGF-β) (Sun et al., 2014). A new study suggested that APS significantly improves cancer symptom clusters in patients with metastatic disease and reduces the expression of major proinflammatory cytokines, including IL1β, IL6, IL12, and IFNγ (Huang et al., 2019).

**Table 1:** Influences of APS on cytokines.

Cytokine	Influence	Model	Reference
IL1β	Upregulation	*In vivo*	(Wang et al., 1989)
	Downregulation	*In vivo*	(Deng et al., 2012; Qiu and Cheng, 2019)
		*In vitro*	(Yuan et al., 2008; Yin et al., 2012)
IL2	Upregulation	*In vivo*	(Shao et al., 2004; Wang et al., 2013; Xie et al., 2013; Yang et al., 2014; Lv et al., 2017)
		*In vitro*	(Liu et al., 2011)
IL3	Upregulation	*In vitro*	(Liu et al., 2011)
IL4	Upregulation	*In vitro*	(Liu et al., 2011)
IL6	Upregulation	*In vivo*	(Lv et al., 2017)
		*In vitro*	(Liu et al., 2011; Li S. et al., 2019)
	Downregulation	*In vivo*	(Deng et al., 2012)
IL8	Downregulation	*In vitro*	(Yin et al., 2012)
IL10	Upregulation	*In vivo*	(Shao et al., 2004; Xie et al., 2013)
	Downregulation	*In vivo*	(Wang et al., 2013; Yang et al., 2014; Liu et al., 2017)
IL11	Upregulation	*In vivo*	(Bamodu et al., 2019)
IL12	Upregulation	*In vivo*	(Wang et al., 2013; Yang et al., 2014)
	Downregulation	*In vivo*	(Li et al., 2018)
IFNγ	Upregulation	*In vivo*	(Liu et al., 2017; Bamodu et al., 2019)
	Downregulation	*In vivo*	(Li et al., 2018)
TGF-β	Upregulation	*In vivo*	(Liu et al., 2017)
TNF-α	Upregulation	*In vivo*	(Wang et al., 1989; Wang et al., 2013; Yang et al., 2014; Liu et al., 2017; Lv et al., 2017; Bamodu et al., 2019)
		*In vitro*	(Li et al., 2019)
	Downregulation	*In vivo*	(Deng et al., 2012; Qiu and Cheng, 2019)
		*In vitro*	(Yuan et al., 2008; Yin et al., 2012)

##### Influences on Ig

The major role of APS in Ig is to mediate immunity through IgA, IgG, and IgM. APS increased the expression of IL2, IL3, IL4, IFNγ, IgM, and IgG, whereas it decreased that of IgE (Lu Y. et al., 2016). Animal experiments affirmed that the production of IgM antibody in aged mice (36 and 60 weeks) was increased following the administration of APS (Kajimura et al., 1997). The level of antibody IgG in the serum of mice infected with Listeria can be significantly increased by APS injection (Xiang et al., 2007). Similarly, the levels of serum IgA, IgM, and IgG in juvenile broilers fed with APS were higher than those reported in broilers without exposure to APS. However, the excessive dose of APS (> 1 g/kg) did not further improve the serum levels of IgA, IgM, and IgG in juvenile broilers (Wu, 2018). The study showed that oral administration of APS promoted the immune function of Newcastle disease-vaccinated chickens and the formation of IgA cells, and increased the secretion of secretory IgA, thus improving mucosal immunity in the jejunum (Shan et al., 2019).

##### Influences on Immune Signal Transduction

**Figure 2:**
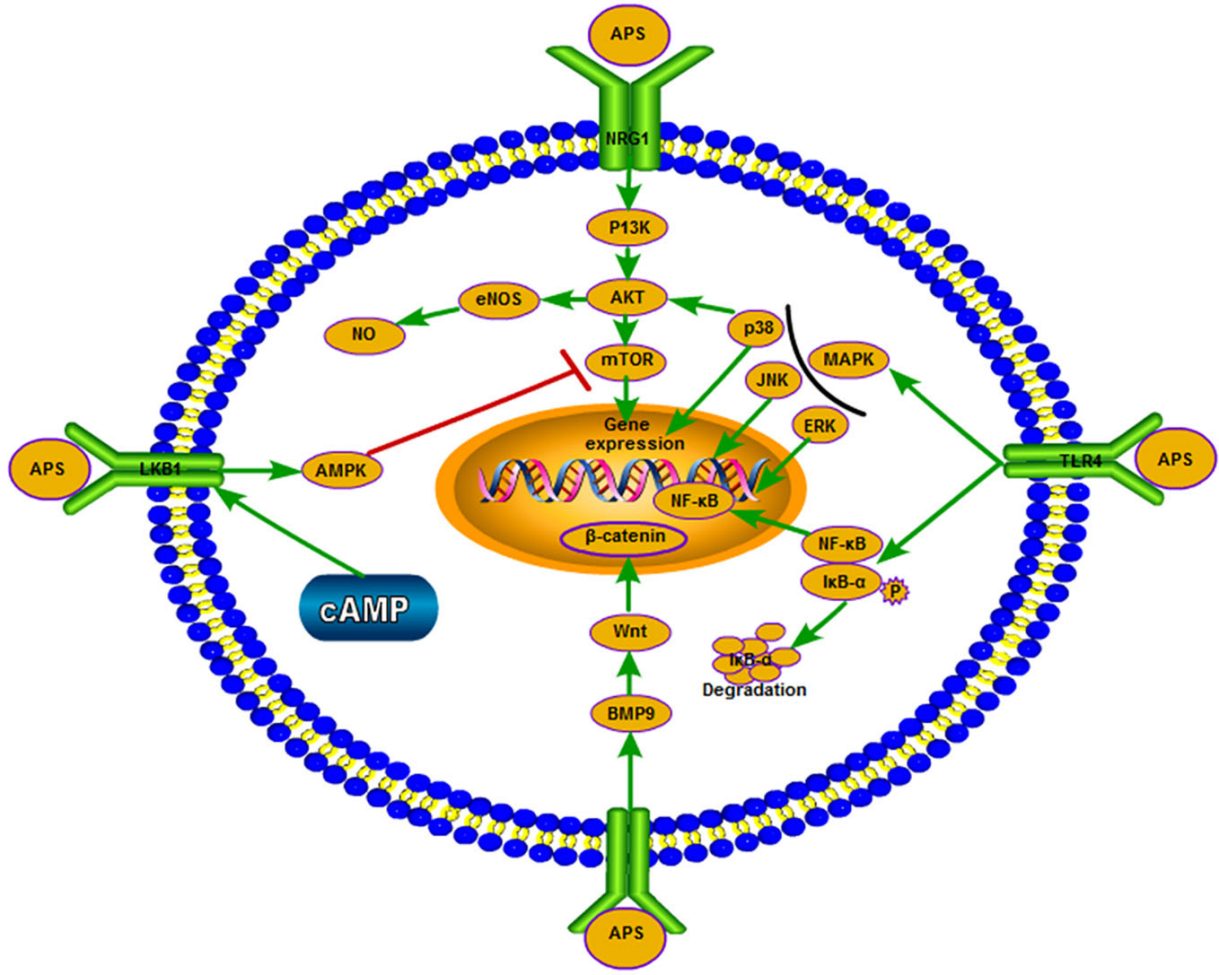
APS exerts different effects on the signaling pathways in different environments and under certain physiological conditions.

Intracellular signal transducers and immune signaling pathways play a key role in the process of immune regulation (**Figure 2**). Studies have shown that APS can increase the TLR4/NF-κB and Ca2+-cAMP signaling pathways in RAW264.7 cells (murine mononuclear macrophage leukemia cells) (Wang Z. et al., 2017). The mouse macrophages is activated by triggering the TLR4-mediated signaling pathways, upregulating the expression of phosphorylated-p38 (p-p38), p-extracellular signal-regulated kinase (p-ERK), and p-JNK, inducing inhibitor of IκB-α degradation and NF-κB translocation, and ultimately enhances nitric oxide and TNF-α (Wei et al., 2016). Recent research has found that APS prevents coxsackievirus B3-induced myocardial injury and inflammation by modulating the TLR4/NF-κBp65 signaling pathway (Liu T. et al., 2019). APS nanoparticles can protect against sepsis-induced cardiac dysfunction by inhibiting the TLR4/NF-κB pathway (Xu et al., 2019). APS supplementation in diet may regulate the immune function of piglets by activating the TLR4-mediated MyD88-dependent signaling pathway (Wang K.L. et al., 2019). APS can also inhibit the expression of thrombin-induced intercellular cell adhesion molecule-1 through blocking the NF-κB signal transduction in rat bone marrow endothelial progenitor cells, and upregulating the expression of vascular endothelial growth factor and its receptor (Zhang et al., 2016). The phosphatidylinositol 3-kinase/protein kinase (PI3K/AKT) signaling pathway regulates cell metabolism, growth, migration, and proliferation. Notably, endothelial nitric oxide synthase is a key enzyme in the regulation of endothelial nitric oxide production, and is regulated by the PI3K/AKT signaling pathway. Following the treatment of H9c2 cells with different concentrations of APS, the result showed that APS protected these cells from LPS-induced inflammatory injury. This effect may also be attributed to the downregulation of miR-127, as well as the adjustment of the JNK, NF-κB, and PI3K/AKT signaling pathways (Ren et al., 2018). APS can promote the proliferation and differentiation of bone marrow mesenchymal stem cells (BMSCs) by upregulating BMP9, during which overexpression of BMP9 activates the PI3K/AKT and Wnt/β-catenin signaling pathways (Li Q. et al., 2019). APS can also partially suppress pulmonary artery remodeling through endothelial nitric oxide synthase/nitric oxide and the NF-κB signaling pathway to improve monocrotaline-induced pulmonary hypertension (Yuan L.B. et al., 2017). In addition, APS can inhibit the expression of adhesion molecules, which is induced by TNF-α through blocking NF-κB signal transduction in human umbilical vein endothelial cells and suppressing the production of reactive oxygen species (Zhu Y.P. et al., 2013). APS activated the downstream PI3K/AKT pathway by inducing neuregulin 1 (NRG1), which enhanced the phosphorylation of PI3K and AKT (Chang et al., 2018). APS can improve muscle atrophy through AKT/mammalian target of rapamycin (AKT/mTOR), autophagy signal transduction, and ubiquitin proteasome; sodium-dependent neutral amino acid transporter (SNAT2) may be one of the latent targets (Lu L. et al., 2016). AMP-activated protein kinase (AMPK) activated by AMP is the most important substrate of liver kinase B1 (LKB1); it is able to sensitively perceive the levels of cellular energy and maintain homeostasis. LKB1/AMPK participates in the regulation of cell growth and cell cycle by regulating mTOR. The mTOR is an important kinase regulating cell growth in eukaryotes. In similar studies, the mTOR inhibitor rapamycin markedly eliminated the protective effect of APS on adriamycin-induced cardiac injury; APS may play a protective role by regulating LKB1/AMPK to regulate mTOR (Cao et al., 2017). In pathological conditions, APS can downregulate the activity of mTOR and protect cells. In a study investigating the effect of APS on iron-overloaded mice, APS activated the p38/mitogen-activated protein kinase (p38/MAPK) signal transduction pathway *in vitro* (Ren et al., 2016). The inhibitory effect of APS on autophagy may be regulated by a mTOR-independent signaling pathway. Moreover, APS reduces hydrogen peroxide-induced apoptosis in myoblast C2C12 cells by inhibiting the Caspase-3 signaling pathway (Yin et al., 2015). By modulating the MEK/ERK pathway to up-regulate Kruppel like factor 2 expression, it can also reduce hydrogen peroxide-induced cell damage in human umbilical vein endothelial cells effectively (Li D.T. et al., 2019). Recent studies have also found that APS can effectively inhibit experimental autoimmune encephalomyelitis-mediated immune response by downregulating proinflammatory cytokines, upregulating the co-stimulatory molecule PD-1/PD-Ls signaling pathway, and inhibiting T cells (Sun et al., 2019).

### Anti-Aging Effect of APS

Numerous studies have found that APS exerts an anti-aging effect through antioxidant and life-prolonging properties. An increase in free radicals *in vivo* can lead to lipid peroxidation. As the end product of lipid peroxide metabolism in cells, malondialdehyde can indirectly reflect the extent of cell damage. And the formation of lipid peroxide can induce the production of antioxidant enzymes in body. If the activity of superoxide dismutase is decreased and the antioxidant ability of the body is decreased, it will lead to attack of free radicals on normal tissues and cells, accelerating the occurrence of aging and diseases. Free radicals can attack normal tissues and cells, as well as accelerate the occurrence of aging and diseases. APS demonstrates resistance to oxidation, and is able to scavenge free radicals, improve oxidative stress, inhibit lipid peroxidation, and chelate iron ions *in vitro* (Pu et al., 2015). In addition, APS increases superoxide dismutase and simultaneously blocks the production of malondialdehyde (Huang et al., 2013); glutathione and total antioxidant capacity can be improved, whereas hydroxyl radical activity can be eliminated (Jia et al., 2012; Tian et al., 2018). APS can play an anti-aging role *via* different mechanisms. It was found that APS significantly prolonged the life span of N2 nematodes in S-complete medium and solid nematode growth medium. It was found that the highly conserved miRNA miR-124 was significantly upregulated in APS-treated nematodes. The results showed that activating transcription factor 6 (ATF6), which is regulated by miR-124, can prolong the life span. This observation may be the basis for the ability of APS to prolong the life span of *Caenorhabditis elegans* (Wang et al., 2015). APS can also exert its anti-aging effects by regulating telomerase activity, regulating or changing telomere-binding proteins, and preventing the shortening of the end of the chromosomal restriction fragment (Zhu G.M. et al., 2013). Furthermore, APS can effectively inhibit the aging of BMSCs induced by ferric ammonium citrate (Yang et al., 2016).

### Antitumor Effect of APS

APS shows its characteristic antitumor properties by enhancing immunity, inhibiting the proliferation of tumor cells, inducing apoptosis of tumor cells, and inhibiting the transfer of tumor cells (**Table 2**).

**Table 2:** Antitumor effect mechanism of APS.

Pharmacological action	Function	Living model	Reference
Inhibition of tumor cell proliferation	Downregulation of cyclin B and cyclin E expression and upregulation of P21 expression	K562 cells	(Jia et al., 2012)
	Blockage of cells in the G0-G1 phase	MKN45 cells HgpG2 cells	(Wang et al., 2015; Tian et al., 2018)
	Decrease in proliferating nuclear antigen expression	COLO205 cells	(Zhu G.M. et al., 2013)
	Cell blockage in the G1 phase; cells in the S phase were obviously reduced	BEL-7404 cells	(Yang et al., 2016)
	Reduction of transcriptional activity of P65mRNA in cells, reduction of LC3B and beclin 1 expression	A549 cells	(Chao et al., 2012; Li, 2014)
	Increase of BAX protein expression, decrease of BCL2 protein expression	H22 cells	(Xie et al., 2009)
		H22 tumor-bearing mice	(Xie et al., 2009)
	Regulation of CDC6 and CCNB1	MCF-7 cells	(Zheng et al., 2012)
Promote tumor cells apoptosis	Cell blockage in the G1 phase; cells in the S phase were obviously reduced	MCF-7 cells	(Zhao and Li, 2005)
		4T1 cells	(Wu et al., 2017)
	Promotion of cell differentiation into the G0-G1 and G0-M phases, decrease in the S phase	HgpG2 cells	(Wang X.L. et al., 2019)
	Inhibition of the ERK1/2 signaling pathway	HgpG2 cells	(Lai et al., 2017)
	Inhibition of the transcriptional activity of NF-κB	A549 cells	(Liu C. et al., 2019)
	Decrease in BCL2 expression, increase in caspase 3 activity	HgpG2 cells	(Li et al., 2019a)
	Increase in the levels of BAX and caspase 8, and decrease in the levels of BCL2	H460 cells	(Li et al., 2019b)
	Downregulation of BAX and BCL2 expression	SGC7901 cells	(Wang, 2012)
	Reduction of telomerase activity	HL-60 cells	(Feng and Zhang, 2010)
	Increase in intracellular calcium concentration	S-180 cells	(Lee and Jeon, 2005)
Inhibit tumor cell metastasis	Decrease in MMP2	C-33A cells	(Wu et al., 2013)
	Up-regulation of E-cadherin expression, inhibition of MMP2	C-4I cells	(Zhang et al., 2015)
	Inhibition of NOTCH1 expression	H22 cells	(Nie, 2014)
	Inhibition of the NF-κB and MAPK signaling pathways	Lewis cells	(Yao et al., 2005)

#### Inhibiting the Proliferation of Tumor Cells

Studies showed that APS can significantly inhibit the proliferation of tumor cells. APS was able to inhibit the proliferation of human erythroleukemia K562 cells by downregulating the expression of cyclin B and cyclin E, as well as upregulating the expression of p21 (Li, 2014). APS also inhibited the growth and proliferation of human gastric cancer cells MKN45 in a dose-dependent and concentration-dependent manner. The mechanism of this process involves blockage of MKN45 cells in the G0-G1 phase by affecting the cell cycle (Xie et al., 2009; Chao et al., 2012). APS at concentrations >50.0 μg/ml inhibited the growth of COLO205 human colon cancer cell lines *in vitro*; the mechanism of this process may be related to a decrease in the expression of proliferating nuclear antigen (Zheng et al., 2012). Other studies have shown that the growth and proliferation of BEL-7404 hepatoma carcinoma cells were significantly inhibited by APS; the cells were blocked in the G1 phase and the number of cells in the S phase was significantly decreased (Zhao and Li, 2005). APS may delay tumor growth of A549 xenografts *in vivo* by reducing the transcriptional activity of P65mRNA in cells (Wu et al., 2017), or inhibit the growth of A549 cells in lung cancer by reducing the expression of LC3B and beclin 1 (Wang X.L. et al., 2019). Research studies revealed that APS inhibited the growth of H22 cells; the mechanism of this process is related to the increase in the expression of BAX protein and decrease in the expression of B-cell lymphoma 2 (BCL2) protein (Lai et al., 2017). Recent research suggested that APS may inhibit the proliferation of MCF-7 breast cancer cells through regulation of control protein 6 homolog (CDC6) and mitotic specific cyclin-B1 (CCNB1) (Liu C. et al., 2019).

#### Promoting the Apoptosis of Tumor Cells

APS is able to significantly promote the apoptosis of tumor cells. Research studies showed that APS can induce the apoptosis of MCF-7 cells and 4T1 cells by blocking the cell cycle in the G1 stage (Li et al., 2019a; Li et al., 2019b). APS may inhibit the ERK1/2 signaling pathway (Wang, 2012) by promoting HepG2 cell differentiation into the G0-G1 and G0-M stages (Feng and Zhang, 2010), inducing the expression of BCL2 in HepG2 cells and increasing the activity of caspase 3 (Lee and Jeon, 2005), thus promoting the apoptosis of HepG2 cells. In a study investigating the apoptosis of human lung cancer A549 cells, it was found that APS may accelerate apoptosis by inhibiting the transcription activity of NF-κB (Wu et al., 2013). Following treatment of human lung cancer H460 cells with APS, the levels of pro-apoptosis BAX and caspase 8 were markedly increased, whereas those of anti-apoptosis BCL2 were decreased; these effects promoted the apoptosis of tumor cells (Zhang J.X. et al., 2015). APS regulates the apoptosis of human gastric cancer SGC7901 cells (Nie, 2014), possibly by downregulating the expression of the p53 downstream genes BAX and BCL2. In addition, the apoptosis of human early childhood leukemia HL-60 cells induced by APS may be induced by decreasing the activity of telomerase (Yao et al., 2005). The apoptosis of mice S-180 sarcoma cells induced by APS may be mediated by increasing the concentration of calcium ions in cells (Luo et al., 2008).

#### Inhibiting the Metastasis of Tumor Cells

APS can significantly inhibit the metastasis of C33A cells in cervical cancer, which may be achieved by reducing the levels of matrix metalloproteinase 2 (MMP2) (Shi, 2014). In addition, APS significantly inhibited the migration and invasion abilities of cervical cancer C-4I cells; this effect may be related to the upregulation of E-cadherin expression and inhibition of MMP2 activity (Chen, 2015). Other studies have shown that APS inhibits the metastasis of hepatocellular carcinoma H22 cells in a concentration-dependent manner and induces apoptosis in H22 cells by inhibiting the expression of NOTCH1 (Huang et al., 2016). Furthermore, APS may inhibit the metastasis of mouse Lewis lung cancer cells by inhibiting the activation of the NF-κB and MAPK signaling pathways (Ming et al., 2016).

### Regulation of Blood Glucose by APS

**Figure 3:**
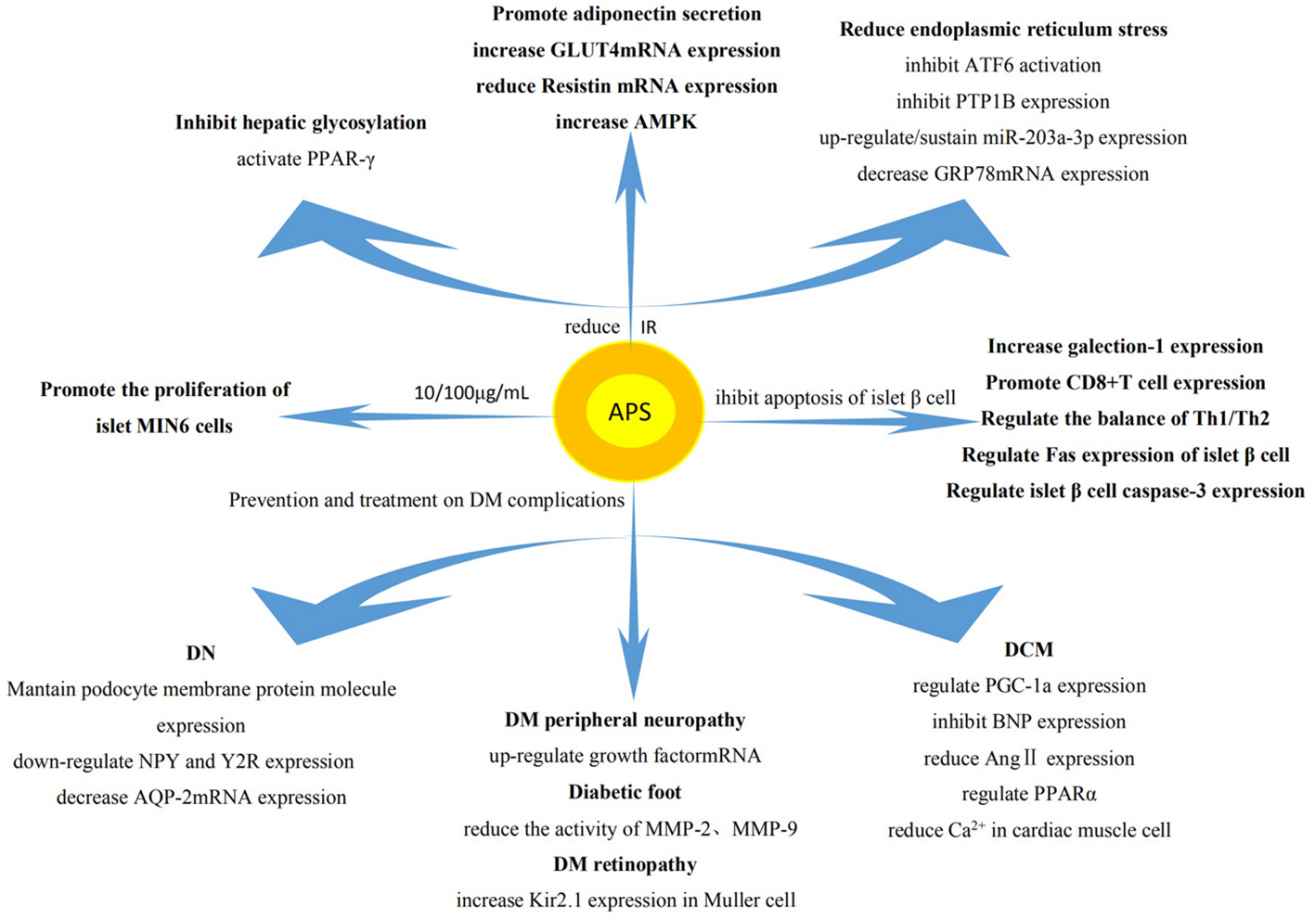
Regulation on blood glucose by APS. The effect of APS on DM mainly involves reduction of IR, promotion of the proliferation of islet cells, and inhibition of the apoptosis of islet β cells. The intrinsic mechanism of its pharmacological action mainly involves influencing the expression of related genes and proteins.

Studies have shown that APS reduces the levels of blood glucose, increases the sensitivity to insulin, improves insulin resistance (IR), and inhibits the apoptosis of islet β cells. It also plays a key role in the treatment of diabetes mellitus (DM) and its complications (**Figure 3**).

#### Effects of APS on Type I Diabetes Mellitus

Type I diabetes mellitus (T1DM) is an autoimmune disease, which mainly leads to the apoptosis of islet β cells and the absolute deficiency of insulin. It has been found that APS can reduce the incidence of T1DM in non-obese diabetic mice, delay the onset of disease, reduce the degree of islet inflammation, and protect the ultrastructure of β cells (Chen et al., 2007). APS also up-regulates the expression of galectin 1 in the muscle of T1DM mice, leading to the apoptosis of CD8+T cells (Zhou et al., 2011). In addition, it regulates the ratio of helper T cell 1 (Th1)/Th2 to restore balance (Jin et al., 2013) and interferes with the expression of Fas and cysteine protease 3 in islet β cells (Li C.D. et al., 2011), thus inhibiting the apoptosis of islet β cells. Moreover, 10 and 100 μg/ml of APS also stimulated the proliferation activity of islet MIN6 cells and decreased the apoptosis of cells, thereby enhancing the production of insulin to lower the levels of blood glucose (Li L. et al., 2011).

#### Effect of APS on Type II Diabetes Mellitus

The pathogenesis of Type II diabetes mellitus (T2DM) is mainly attributed to IR, the functional defect of islet β cells, and the relative insufficiency of insulin. The normal functions of islet β cells including decreasing IR, stabilizing lipid metabolism, increasing the secretion of insulin, and reducing the levels of blood glucose (Song et al., 2013). APS can also protect islet β cells, reduce the levels of blood glucose and fasting insulin in T2DM rats effectively (He and Zhu, 2018). Some studies showed that APS can reduce blood glucose by reducing endoplasmic reticulum stress in patients with T2DM, thereby increasing the sensitivity to insulin (Hu et al., 2010). Its mechanism may involve reduction of the activation of ATF6 (induced by endoplasmic reticulum stress), reversal of the translocation of ATF6 in cells, inhibition of the high expression of protein tyrosine phosphatase 1B (PTP1B). These effects relieve endoplasmic reticulum stress in the liver to produce the sensitizing effect of insulin (Wang et al., 2009). Moreover, APS may inhibit the IR of T2DM by upregulating or maintaining the expression of miR-203a-3p, decreasing the expression of glucose-regulated protein (GRP78) mRNA and protein, and regulating the expression of protein in the signaling pathway of endoplasmic reticulum stress (Wei et al., 2017). APS can also bind and activate peroxisome proliferator-activated receptor γ, significantly inhibit hepatic glycosylation, increase the non-oxidative metabolism of glucose in skeletal muscle, and significantly improve IR in patients (Seino et al., 2007). It also promotes cell differentiation, induces the secretion of adiponectin (Hu et al., 2018), increases the expression of T2DM rat skeleton (Liu et al., 2011a) and glucose transporter 4 (GLUT4) mRNA in adipose tissue (Liu et al., 2011b), decreases the expression of resistin mRNA, downregulates adipose tissue resistance protein in T2DM rats (Liu et al., 2012c), and increases the expression of AMPK in liver tissue of T2DM rats (Wu et al., 2009). Collectively, these effects improve IR.

#### Effect of APS on Diabetic Complications

APS can prevent and treat other chronic complications of DM, such as diabetic nephropathy (DN), diabetic cardiomyopathy (DCM), etc.

##### Influence of APS on DN

DN is a common and serious chronic complication of DM. It is an important cause of death in patients with DM. The protective effect of APS on DN may be related to maintaining the expression of nephrin and podocin in podocytes (Li Z.J.et al., 2011). Moreover, it may relevant to downregulating the expression of renal neuropeptide y and its Y2 receptor (Chen et al., 2011). The mechanism of APS in the treatment of DN may be related to the reduction of the expression levels of renal medullary aquaporin 2 (AQP2) mRNA (Mao et al., 2010).

##### Influence of APS on DCM

DCM, a chronic complication of DM, is a specific cardiomyopathy that occurs in the absence of coronary artery disease and hypertension (Bai et al., 2016). Treatment with APS can improve the apoptosis and necrosis of cardiac muscle cells in diabetic rats (Chen et al., 2018). The mechanism involved in this process may reduce the apoptosis of cardiac muscle cells in diabetic rats by affecting the expression of peroxisome proliferator-activated receptor γ coactivator 1α (Yu et al., 2015) or by inhibiting the expression of brain natriuretic peptide (Wang F. et al., 2012). APS may also exert a protective effect on DM cardiomyopathy by decreasing the expression of myocardial angiotensin II (ANG II) and inhibiting the production of regional chymase-dependent ANG II in the diabetic cardiac muscle (Chen et al., 2010). Moreover, it prevents the development of lipotoxic cardiomyopathy by regulating PPARα (Chen et al., 2015). Furthermore, overload of intracellular calcium is also the cause of DCM; APS can reduce intracellular calcium overload and alleviate myocardial dysfunction (Liu et al., 2011d).

##### Influences of APS on Other Chronic Complications of DM

Studies have shown that the expression of brain nerve growth factor mRNA decreased in T2DM rats. However, it increased in T2DM rats after the administration of APS (Liu et al., 2011e), indicating that APS may be able to improve diabetic peripheral neuropathy. APS can also alleviate the increase in MMP2 and MMP9 activity and their protein expressions in fibroblasts from diabetic foot ulcer, thus promoting healing (Zhang et al., 2007). It also reduces the incidence of DM retinopathy by reversing the decrease of Kir2.1 protein expression in early retinal Muller cells of DM rats (Li et al., 2008).

### Other Pharmacological Effects of APS

#### Antiviral Effect of APS

An *in vitro* experiment revealed that APS inhibited the replication of porcine circovirus type 2 by reducing oxidative stress and activation of the NF-κB signaling pathway (Xue et al., 2015). APS also inhibited the proliferation of astrocytes infected by the herpes simplex virus (Shi et al., 2014). In addition, it effectively inhibited the cytopathic changes induced by coxsackievirus B3 and the proliferation of the virus in Vero cells and cardiomyocytes (Liu et al., 2003). It was also found that APS at a non-cytotoxic concentration of 30 μg/ml significantly inhibited the expression of zipper transcription factor, transcription activator, and the diffuse component of early antigen in the Epstein-Barr virus cleavage cycle, showing that APS carries potential as an anti-Epstein-Barr virus drug (Guo et al., 2014). Moreover, APS inhibited the replication of infectious bronchitis virus *in vitro*. The observed decrease in viral replication after treatment with APS was related to the decrease of cytokines; these results showed that APS had activity against infectious bronchitis virus (Zhang et al., 2018). Treatment with APS also reduced the replication of H9N2 avian influenza virus (AIV) and promoted the early humoral immune response in young chicken, thus enhancing the cellular immunity (Sanpha et al., 2013).

#### Antagonistic Fibrosis Effect of APS

It has been found that APS reduced the production of collagen in skin treated with bleomycin, and was able to antagonize prostaglandin-induced fibrosis (Hao et al., 2015). APS may prevent and treat myocardial fibrosis induced by isoproterenol by inhibiting the TGF-β1/SMADs pathway (Zhu et al., 2017). APS also inhibited the formation of renal interstitial fibrosis and protected the kidney to some extent by upregulating the expression of MMP2 and downregulating that of TGF-β1, tissue inhibitor of MMP1, and ANG II in renal interstitial tissue (Lu and Wei, 2014). APS may also prevent and treat hepatic fibrosis by inhibiting the expression of type I collagen and α-smooth muscle actin in rats (Zhang C. et al., 2015).

#### Lowering Blood Lipids Effect of APS

It has been found that APS can improve hyperlipidemia in mice (Liu et al., 2013). It decreases the levels of serum triglycerides in T2DM rats and improves the disorder of body fat metabolism (Luo et al., 2007). APS can significantly reduce the levels of serum leptin in obese rats induced by high-fat diet, by increasing the levels of serum adiponectin (Chen et al., 2013). The content of total cholesterol, triglycerides, and low-density lipoprotein cholesterol in the blood of rats with non-alcoholic fatty liver disease was significantly decreased after treatment with APS. The results showed that APS decreased the levels of blood lipids and retarded the accumulation of fat in the liver of rats with fatty liver (Tang et al., 2016). By comparing the therapeutic effects of APS and simvastatin on hyperlipidemic rats, it was found that APS exerted a similar lipid-lowering effect to that of simvastatin after prolonging the administration time, and was superior to simvastatin in improving the effect of transaminase (Yuan Q.F. et al., 2017).

#### Antimicrobial Effect of APS

It was found that APS exerted a certain bacteriostatic effect on the main pathogenic bacteria causing mastitis in dairy cattle, including *Streptococcus*, *Escherichia coli*, and *Staphylococcus aureus*; this bacteriostatic effect was dose-dependent (Yang et al., 2010). At concentrations of 20 mg/L and 40 mg/L, APS also significantly inhibited the bacterial strains of *Staphylococcus aureus*, *Escherichia coli*, and *Salmonella in vitro* (Li et al., 2007). The water-soluble portion of the polysaccharide extracted from the root of *A. membranaceus* was used to synthesize silver nanoparticles (AgNPs). The results showed that *A. membranaceus*-mediated AgNPs was resistant to clinical multidrug-resistant bacteria (*Methicillin-resistant S. aureus*, *Methicillin Resistant S. Epidermidis*, *E. coli*, *P. aeruginosa*), and showed significant antibacterial activity and relatively low concentrations of reference strains (*S. aureus* ATCC 29213, *S. epidermidis* ATCC 12228, *E. coli* ATCC 25922, *P. aeruginosa* ATCC 15442). In particular, APS demonstrated great potential for eliminating multidrug-resistant bacteria (Ma et al., 2017).

#### Radiation Protection Effect of APS

It was found that APS could alleviate DNA damage in BMSC after X-ray irradiation (Zhou et al., 2016), as well as damage to the nucleus and chromosomes (Wang et al., 2008). APS may also prevent the injury to interstitial glands induced by 60coγ-ray by promoting the expression of luteinizing hormone receptor mRNA in Leydig cells of the testes (Wang R. et al., 2012). It was found that the compatibility of angelica polysaccharide and APS at a 3:1 ratio significantly reduced damage to model mice caused by radiation (Ding et al., 2014). The compatibility of APS and Astragaloside A at a 4:1 ratio also markedly reduced radiation-induced damage in model mice (Liu et al., 2014).

## Conclusion and Future Perspectives

This article described the nine main pharmacological effects of APS. The main function of APS is to promote repair and regulation of the immune system. However, there are few reports on the modification of the APS structure. Thus, future research studies should investigate whether different APS modifiers can enhance its immune regulatory function. The mechanism of antioxidation is the most important in the anti-aging effect of APS, which is generally considered to be effective in the prevention and cure of cancer. Notably, there is usually abnormal energy metabolism in tumor cells. However, there are few reports investigating whether APS can regulate the energy metabolism in tumor cells to exert its characteristic antitumor activity. Hence, this is also one of the research directions regarding the antitumor effect of APS. APS offers great advantages in the treatment of DM and its complications. As a natural drug, it is suitable for long-term use in patients with chronic disease. Therefore, it is important to elucidate the mechanism of APS involved in the treatment of DM. Nevertheless, further clarification of its mechanisms involved in reducing blood lipids, antagonizing fibrosis, bacteriostasis, radiation protection, and antiviral activities is warranted. APS, as the extract of *A. membranaceus*, exhibits almost no toxic side effects. The dose-response relationship is an important factor in the prevention and treatment of diseases. Thus, the precise control of the dosage of APS, while exerting its various pharmacological effects, requires further investigation. APS has great potential in the treatment of diseases. At present, injection with APS has been used to assist in radiotherapy and chemotherapy, and play a synergistic role in reducing toxicity. APS is a natural complex compound; thus, the greatest current challenge in APS research is to extract its specific components and identify their precise targets. Accurate detection of the targets of the nine pharmacological actions of APS and demonstration of the remarkable effects of the multi-target integration of traditional Chinese medicine will be more instructive for the clinical use of APS.

## Author Contributions

DL and YL organized thoughts for the article and revised the manuscript. YJZ and WR were responsible for the writing of the article and the inquiry of the information. LNZ translated and revised the article. YMZ offered opinions for the drawing of diagrams and charts in the article. All authors read and approved the final version of the manuscript.

## Funding

This project was supported by the Natural Science Foundation in China (grant numbers 81603407, 81973595).

## Conflict of Interest

The authors declare that the research was conducted in the absence of any commercial or financial relationships that could be construed as a potential conflict of interest.
